# Neuropsychological Function and the Relationship Between Subjective Cognition, Objective Cognition, and Symptoms in Hypermobile Ehlers–Danlos Syndrome

**DOI:** 10.1002/brb3.70603

**Published:** 2025-05-30

**Authors:** Amber Sousa, Min‐Kyung Jung, Arline Allera, Bernadette Riley

**Affiliations:** ^1^ New York Institute of Technology (NYIT) College of Osteopathic Medicine Old Westbury New York USA

**Keywords:** cognition, hypermobile Ehlers‐Danlos syndrome, memory, neuropsychology, subjective cognition

## Abstract

**Introduction:**

Hypermobile Ehlers–Danlos syndrome (hEDS), a subtype of the Ehlers–Danlos syndromes, is a connective tissue disorder that is associated with a number of cognitive and psychological symptoms. Very little research has directly examined the neuropsychological functioning profile in hEDS, but some research has found associations of hypermobility with cognitive difficulties, psychological symptoms, and structural brain differences.

**Methods:**

The current research compared a 12‐matched‐pairs sample of individuals with hEDS to healthy controls on a comprehensive neuropsychological test battery, as well as measures of mood, pain levels, fatigue, subjective cognition, and ability to perform social activities. Participants were matched for age, gender, and years of education. A second analysis was completed for a larger group of 18 participants with hEDS to examine associations of cognition and other symptoms with subjective cognition.

**Results:**

Our results did not reveal significant cognitive differences between the two groups for most cognitive measures. However, individuals with hEDS had lower scores for delayed verbal memory recall. In the larger hEDS sample, correlations between subjective cognition, objective cognitive test performance, and other clinical variables revealed significant correlations between subjective cognition and visuospatial planning and construction, working memory, and set‐shifting. Additionally, subjective cognition was associated with anxiety, depression, fatigue, pain, and the ability to participate in social activities.

**Conclusion:**

We conclude that cognitive difficulties reported by individuals with hEDS are likely fluctuating and may correspond with fluctuating symptoms including dysautonomia, pain, and sleep difficulties. Furthermore, subjective cognition appears to be especially correlated with other related symptoms of mood, pain, and fatigue.

## Graphical abstract

Neuropsychological function in hypermobile Ehlers–Danlos syndrome (hEDS) was found to be characterized by globally intact cognition with weaker delayed verbal memory. People with hEDS reported more symptoms of depression, fatigue, pain, subjective cognitive difficulty, and trouble participating in social roles. Subjective cognition was especially tied to social participation.

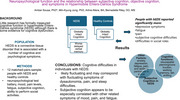

## 1 Introduction

The Ehlers–Danlos syndromes (EDS) are a group of heritable connective tissue disorders that affect the body in various ways, including hypermobility, chronic pain, hyperextensibility, and tissue fragility (Bloom et al. 2017). The hypermobile type of Ehlers–Danlos syndrome (hEDS) is reported to be the most common type of EDS. It is considered a rare disorder with an estimated prevalence rate of 1 in 5000; however, due to varying definitions and symptom overlap with generalized joint hypermobility syndrome, this prevalence rate remains unclear and reliant on varying diagnostic criteria (Riley 2020). Adding to this uncertainty, although hEDS is observed to typically follow an autosomal dominant familial genetic pattern, there is no clearly implicated gene that accounts for any significant proportion of hEDS cases (Tinkle et al. 2017).

In addition to the characteristic physical components of hEDS, there are a number of reported psychological, cognitive, neurological, and psychosocial aspects of, or associations with, the disease (Kennedy et al. 2022). These include mood disorder, depression, anxiety, panic disorder, personality disorders, autism spectrum disorder, sleep disturbance, cognitive complaints, and measurable cognitive deficits (Pasquini et al. 2014; Cederlof et al. 2016; Baeza‐Velasco et al. 2018). While the underlying etiologies of these additional manifestations in the context of hEDS are not fully understood, there is evidence that hypermobility disorders may also coincide with neurological differences, chronic pain, and a constellation of symptoms. Structural brain differences have been found in individuals with hypermobility. Specifically, individuals with non‐clinical hypermobility were found to have significantly larger amygdala compared to non‐hypermobile individuals. Degree of hypermobility also correlated positively with left lateral occipital cortex volume and negatively with right superior temporal cortex and bilateral inferior parietal cortices (Eccles et al. 2012). Associations between hEDS and neuropathy, epilepsy, and motor developmental problems have also been reported in the literature (Castori and Voermans 2014).

Cognitive functioning in hEDS has received very little research attention, presumably because hEDS has not historically been seen as a disease with direct neurological effects. However, cognitive differences were found in people with hEDS in one study. A brief neuropsychological screening measure revealed these cognitive weaknesses to include visuospatial problem solving, attention, memory, and awareness of performance. The same study found no significant relationship between pain intensity and cognitive function but did find a trend‐level relationship between fatigue and cognition, such that lower cognitive performance was observed in patients with severe fatigue. The authors also noted that the cognitive deficits measured in hEDS patients were not related to depressive symptoms, as the participants had been screened for such symptoms (Baeza‐Velasco et al. 2017). However, this screening excluded only those who met criteria for a depressive disorder but did not examine subjects' symptoms of depression or other psychiatric symptoms as they relate to cognition. Therefore, the relationship between cognition and psychiatric symptoms has not been fully explored in the hEDS population.

Cognitive functioning may be measured objectively by neuropsychological tests or subjectively as an individual's perceptions of their own cognition. A lack of association between subjective and objective cognition has been shown in various patient populations, including healthy individuals and people with affective disorders (Svendsen et al. 2012; Crumley et al. 2014; Groenman et al. 2022). Specifically, individuals with depression have been shown to underestimate their own cognition, while healthy controls overestimated their own cognition (Schwert et al. 2018). Others have found that subjective cognition is more associated with mood state than with objective cognitive performance (Marino et al. 2009). These findings highlight the need for both objective neuropsychological assessment and subjective cognitive rating.

Cognitive functioning, both objective and subjective, has been found to be associated with frequent symptoms in hEDS, including psychiatric symptoms, fatigue, and pain. Meta‐analyses have found cognition, especially reduced executive function, attention, processing speed, and memory, to be associated with pain (Moriarty et al. 2011; Berryman et al. 2014; Nadar et al. 2016; Eizaguirre et al. 2020). A comprehensive review found cognitive impairments, especially in executive function, are common in young adults with anxiety and depressive disorders (Castaneda et al. 2008). In a systematic review of the relationship between fatigue and cognition in chronic diseases, while some studies found associations, there was no clear definitive picture of the relationship between the two constructs (Menzies et al. 2021). In people with hEDS specifically, the limited existing research indicates that the relationship between chronic pain and cognitive and emotional functioning seems to be complex and multifaceted (Voermans et al. 2010; Baeza‐Velasco et al. 2019).

Characterizing the neuropsychological profile in individuals with hEDS and related clinical factors has the potential to improve treatments for this disease and associated symptoms and conditions. This information would also pave the way for clinical trials of interventions, including cognitive training and cognitive strategy implementation. These potential treatments would have the capacity to improve affected individuals' daily life functions and quality of life.

## 2 Methods

### 2.1 Participants

The current study was approved on September 10, 2019, by the New York Institute of Technology (NYIT) Institutional Review Board and reviewed on a continuing basis. This was an observational cross‐sectional comparison of a group of individuals diagnosed with hEDS compared with a group of controls matched for age, gender, and education (years). Participants were between the ages of 18 and 45 years and largely recruited from the NYIT‐College of Osteopathic Medicine Ehlers–Danlos Syndrome Center, Old Westbury, New York, between January 26, 2021 and March 7, 2023. Patients with hEDS were recruited if they had a documented diagnosis consistent with the 2017 International criteria (Malfait et al. 2017). Exclusion criteria included any diagnosis of another form of EDS or connective tissue disorder, current pregnancy or perimenopause, and any neurological or musculoskeletal disorder or any disorder known to affect cognition. Healthy controls were community‐recruited people with no history of hEDS, age‐matched within 3 years of affected individuals, education‐matched within 2 years of affected individuals, and gender‐matched, with the same exclusion criteria. Matching variables were chosen due to their known associations with cognitive and psychological function.

### 2.2 Procedures

Procedures were carried out via Zoom sessions with screen‐sharing and the use of computerized links. All participants first underwent the informed consent process with the principal investigator. Following verbal and written informed consent, demographic information was collected via a secure RedCap database. A comprehensive battery of neuropsychological measures was completed with an experienced neuropsychologist via Zoom, utilizing verbal administration, screen‐sharing of stimuli, and computerized links for remotely administered measures. Table 1 lists the measures administered. Breaks were given as needed to prevent fatigue, frustration, and pain. Additionally, participants were tested at their subjectively reported “best” cognitive time of day, and if acute pain, illness, or discomfort were noted, participation was rescheduled. Participants then completed questionnaires measuring mood, pain, fatigue, subjective cognitive function, and ability to participate in social activities. Procedures were largely completed in a single‐day session.

**TABLE 1:** Neuropsychological measures administered and associated outcome variable name.

Domain	Test name	Description
*Attention and executive function*	Integrated Visual and Auditory Continuous Performance Test (IVA‐2)	A measure of sustained auditory and visual attention whereby the subject clicks the computer mouse when they either hear or see a specified number but not when they hear or see other numbers. The global quotient for sustained attention for combined auditory and visual information was used for analyses (Tinius 2003).
	Wechsler Memory Scale‐Revised Digit Span (longest span correct)	A measure of simple auditory attention and working memory whereby the subject repeats increasingly longer strings of digits in the forward and backward direction (Wechsler 1987).
	Trail Making Test Part A and B (milliseconds)	A speeded measure of visual scanning and attention and a separate measure of mental flexibility. The subject is presented a page of disorderedly placed numbers and asked to connect them in order as quickly as possible, and then is presented with a page of disorderly placed numbers and letters and asked to switch between numbers and letters in numerical and alphabetical order (Germine et al. 2012).
	NIH toolbox list sorting working memory	A measure of working memory whereby the subject is presented with a list of pictured items on an iPAD through screenshare and asked to recite them in size order (Weintraub et al. 2013).
*Memory function*	Rey Auditory Verbal Learning Test	A measure of verbal memory requiring subject to recite the list of words after each learning trial and subsequently recall the words after 30‐min delay (Schmidt 2004).
	Rey Osterrieth Complex Figure Test	A measure of spatial memory for a complex abstract figure. Subject first copies the figure, then draws it from memory, and finally draws it once more at 30 min delay (Rey 1941; Osterrieth 1944).
*Language function*	Delis–Kaplan executive function system phonemic fluency	A measure of language requiring the subject to name as many words as they can generate beginning with a particular letter for 1 minute (Delis et al. 2001).
	Delis–Kaplan executive function system semantic fluency	A measure of language requiring the subject to name as many words as they can generate belonging to a specified category (Delis et al. 2001).
	NIH toolbox picture vocabulary	A measure of semantic knowledge whereby subjects choose an image from four choices for which best describes words of increasing difficulty (Gershon et al. 2010, 2001).
*Mood, pain, fatigue, daily function and subjective cognition inventories, and scales*	Patient Reported Outcomes Measurement Information System (PROMIS)(30) Short From v1.0‐ Depression 8b	An 8‐item self‐report measure of depressive symptoms measured by Likert scale format (Pilkonis et al. 2014).
	PROMIS Short Form v1.0‐ Anxiety 8a	An 8‐item self‐report measure of anxiety symptoms measured by Likert‐scale format (Pilkonis et al. 2014).
	PROMIS v1.0 Pain Intensity Scale	A 3‐item self‐report measure of pain intensity for the past week and current time (Amtmann et al. 2010).
	PROMIS Short From v1.0‐ Fatigue 8a	An 8‐item self‐report measure of fatigue in the past week measured by Likert‐scale format (Lai et al. 2011).
	PROMIS Short Form v2.0‐ Cognitive function abilities 8a	An 8‐item self‐report measure of cognitive functions in a Likert‐scale format (Lai et al. 2014).
	PROMIS Short Form v2.0‐ Ability to participate in social roles and activities 8a	An 8‐item self‐report inventory of amount of limits to roles in Likert‐scale format (Hahn et al. 2014).

### 2.3 Data Analysis

Analyses were conducted using IBM SPSS version 28.0.1.0. First, the two groups were compared using matched—pairs *t*‐tests for demographic variables. Because groups were unequal for race, we controlled for race in all subsequent analyses. For all comparison variables, a univariate general linear model (ANOVA) was utilized to compare hEDS participants and controls while controlling for the effects of race. Statistical significance was determined by citing a significance level of 0.05 after adjusting the *p*values with the multiplicity correction procedure of Benjamini–Hochberg (Benjamini and Hochberg 1995). Finally, data from a larger sample of participants with hEDS, which included the 12 hEDS participants from the initial analyses, were examined for correlations of subjective cognition with objective cognition, mood, pain, fatigue, and social participation. Spearman's rho was computed for the correlation, and its strength was interpreted based on Cohen's guideline: 0.1 for weak correlation, 0.3 for moderate correlation, and 0.5 for strong correlation (Cohen 1988).

## 3 Results

For the matched‐pair analyses, 12 individuals with hEDS were compared to 12 matched controls. Table 2 illustrates the demographic variables used for matching. Groups were unequal for race. There were no significant differences between the hEDS participants and controls for age, education, or gender. The average age for the overall sample was 27.0 years, the average years of education was 16.3, and 11 of 12 participants in each group identified as female, while one of each group identified as male.

**TABLE 2:** Demographic variables for matched‐pairs groups.

Variable	Group		*p* value
	hEDS	Controls	
***N***	12	12	
**Mean (SD)**			
Age	27.5 (6.3)	26.6 (5.7)	0.73
Education (years)	15.6 (2.0)	17.0 (2.1)	0.17
**Frequency (%)**			
Gender			
Female	11 (91.7%)	11 (91.7%)	1.00
Male	1 (8.3%)	1 (8.3%)	
Race			
White	11 (91.7%)	3 (25.0%)	0.002
Hispanic/Latino	1 (8.3%)	1 (8.3%)	
Asian	0 (0.0%)	8 (66.7%)	
Occupational status			
Full‐time	8 (66.7%)	11 (91.7%)	0.18
Part‐time	1 (8.3%)	1 (8.3%)	
Unemployed or student	3 (25.0%)	0 (0.0%)	

Across all objective cognitive ability measures examined, which included domains of memory, attention, executive functioning, language, and visuospatial functioning, there was one significant difference between the hEDS group and control group. This was for delayed memory free recall for a list of words. Participants with hEDS recalled fewer words after a delay (*p* = 0.010, *η* ^2^ = 0.29). Table 3 presents these results. On measures of self‐reported symptoms, the hEDS group endorsed significantly more depressive symptoms (*p* = 0.014, *η* ^2^ = 0.27), fatigue (*p* = 0.003, *η* ^2^ = 0.35), pain (*p* = < 0.001, *η* ^2^ = 0.53), subjective cognitive difficulties (*p* = 0.015, *η* ^2^ = 0.26), and difficulty participating in social roles and activities (*p* = 0.003 *η* ^2^ = 0.37). Table 3 also presents these results.

**TABLE 3:** Comparison of outcome measurement scores for hEDS and controls.

Variable	Group		*p* value
	hEDS	Controls	
**Attention and executive function**			
Trail Making Test Part A	24,750.5 (2150.2)	24,208.1 (2266.5)	0.89
Trail Making Test Part B	35,413.9 (5894.4)	36,539.6 (6213.2)	0.90
Digit span forward	6.7 (0.7)	7.2 (0.5)	1.00
Digit span backward	6.4 (0.7)	5.9 (0.4)	1.00
NIH toolbox list sort working memory	17.5 (1.3)	20.2 (0.9)	0.60
IVA‐2 sustained attention	70.5 (18.8)	74.4 (12.0)	0.86
**Memory function**			
Rey Auditory Verbal Test—Total recall	47.0 (3.8)	56.2 (2.4)	0.08
Rey Auditory Verbal Test—Delayed recall	8.0 (1.3)	12.4 (0.8)	0.04
Rey Complex Figure Test—Visuospatial copy	35.6 (1.3)	32.2 (4.3)	0.09
Visual learning—Delayed recall	29.0 (3.7)	20.3 (2.4)	0.06
**Language function**			
D‐K Verbal Fluency—Phonemic	36.9 (6.1)	41.2 (3.9)	1.00
D‐K Verbal Fluency—Switching	15.1 (1.3)	15.9 (0.8)	0.83
D‐K Verbal Fluency—Semantic	42.8 (5.2)	43.9 (3.3)	0.85
NIH Toolbox Picture Vocabulary	84.7 (11.9)	60.0 (7.6)	0.40
Mood, pain, fatigue, daily function, and subjective cognition			
Depression *t*‐score	62.1 (4.2)	48.7 (2.7)	0.021
Anxiety *t*‐score	59.5 (4.0)	51.2 (2.6)	0.10
Pain *t*‐score	53.4 (3.4)	34.2 (2.2)	< 0.001
Fatigue *t*‐score	60.0 (3.7)	45.6 (2.4)	0.009
Cognitive function *t*‐score	41.8 (3.3)	52.3 (2.1)	0.018
Social participation ability *t*‐score	37.2 (4.9)	57.0 (3.1)	0.006

*Note*: Reported are the race‐adjusted mean (SE), and the *p* value from a factorial analysis of variance with Benjamini–Hochberg procedure to control the false discovery rate per experiment.

Finally, in our larger sample of 18 individuals with hEDS, bivariate Spearman's rank correlations were completed for subjective cognitive ability with all objective cognitive neuropsychological measures and all self‐report measures including mood, pain, fatigue, and ability to participate in social roles and activities. Demographics for the larger group are presented in Table 4. For this group, the average age was 29.1 years, the average years of education was 15.8 years, 16 of 18 (88.9%) were white race, 16 of 18 (88.9%) identified as female, and 12 of 18 (66.7%) were employed full‐time. Correlations are presented in Table 5. Moderate correlations were found between subjective cognition and performance on a measure of working memory (digit span backward, *rs* = 0.36), visuospatial copy (Rey Complex Figure Test copy, *rs* = 0.44), executive functioning (D‐KEFS verbal fluency switching, *rs* = 0.40), where more subjective cognitive difficulties were associated with worse performance on those measures. Moderate correlations were also detected for depression (*rs* = −0.33), anxiety (*rs* = −0.44), fatigue (*rs* = −0.38), and pain (*rs* = −0.42), with worse subjective cognition associated with more symptoms. Finally, there was a strong correlation between subjective cognition and ability to participate in social roles, with worse subjective cognition associated with more difficulty in social role participation (*rs* = 0.51).

**TABLE 4:** Demographic variables for larger hEDS participant sample.

Variable	Group
	hEDS
*N*	18
Mean (SD)	
Age	29.1 (7.4)
Education (Years)	15.8 (2.6)
Frequency (%)	
Gender = Female	16 (88.9%)
Race = White	16 (88.9%)
Occupational Status = Full‐time	12 (66.7%)

**TABLE 5:** Correlation of subjective cognitive ability with objective cognitive variables and subjective mood, pain, fatigue, and social participation ability in hEDS participants.

Correlation of subjective cognitive ability with	*N*	Spearman's rho	Strength
**Attention and executive function**			
Trail Making Test Part A	15	0.15	Weak
Trail Making Test Part B	15	−0.08	Weak
Digit span forward	18	−0.25	Weak
Digit span backward	18	0.36	Moderate
NIH toolbox list sort working memory	17	−0.03	Weak
IVA‐2 sustained attention	18	−0.18	Weak
**Memory function**			
Rey Auditory Verbal Test—Total recall	18	−0.26	Weak
Rey Auditory Verbal Test—Delayed recall	18	−0.10	Weak
Rey Complex Figure Test—Visuospatial copy	18	0.44	Moderate
Visual learning—Delayed recall	18	0.20	Weak
**Language function**			
D‐K Verbal Fluency—Phonemic	18	0.20	Weak
D‐K Verbal Fluency—Switching	18	0.40	Moderate
D‐K Verbal Fluency—Semantic	18	0.28	Weak
NIH Toolbox Picture Vocabulary	17	0.09	Weak
**Mood, Pain, Fatigue, Daily Function and Subjective Cognition**			
Depression *t*‐score	18	−0.33	Moderate
Anxiety *t*‐score	18	−0.44	Moderate
Fatigue *t*‐score	18	−0.38	Moderate
Pain *t*‐score	18	−0.42	Moderate
Social participation ability *t*‐score	18	0.51	Strong

*Note*: The value (rho) of the Spearman's correlation coefficient was interpreted using the Cohen's guideline to be a weak correlation for rho < 0.3, a moderate correlation for 0.3 ≤ rho < 0.5, and a strong correlation for 0.5 ≤ rho.

## 4 Discussion

This study compared neuropsychological functioning between a group of people with a hEDS diagnosis and an age, gender, and education‐level matched control group of people who had no history of EDS, connective tissue disorder, musculoskeletal condition, or known cognitive disorders. No prior study to date was found in the published literature that comprehensively examined neuropsychological functions specifically in the context of this rare disease. We found that the hEDS group and control group were significantly different on the demographic variable of race and we therefore corrected for race throughout the analyses. Across all of the cognitive variables, there was only one significant difference in objective cognitive measures, which was for memory for a list of words after a delay.

The lack of significant differences between a group of people with hEDS and matched controls across a large battery of objective cognitive tests was a somewhat unexpected finding, given the frequency of cognitive concerns in the clinical population (Fairweather et al. 2023). Our methods of testing participants at their self‐identified “best” time cognitively allowed us to determine that cognition, at its best, in people with hEDS is not generally impaired or significantly different for most measures, despite a significant level of subjective cognitive difficulty in this sample. We speculate that the cognitive difficulties noted by many of the participants with hEDS may be transient, fluctuating with other factors, and do not represent indicators of permanent or progressive cognitive loss or impairment. However, it is important to note that daily cognitive function in “real‐life” situations are likely impacted by a number of factors that are better controlled during standardized cognitive assessments (e.g., testing at the best cognitive time of day, taking breaks as needed, limiting distractions, and testing at a lower pain time).

The significant finding of delayed verbal free recall memory being significantly worse in people with hEDS is consistent with findings from the limited research that has examined cognition in people with hEDS and joint hypermobility syndrome. These authors found that women with these syndromes had lower scores for memory, attention, and visuospatial problem‐solving (Baeza‐Velasco et al. 2017). Furthermore, our finding that subjective cognition correlated with some areas of objective cognition does signify that people with hEDS are identifying tangible cognitive performance.

It is evident that people with hEDS experience symptoms known to affect cognition negatively, including chronic pain, sleep difficulty, fatigue, dysautonomia, among other factors. We surmise that people's subjective difficulties may be partly related to one or more of these factors. There is evidence for fluctuation in cognitive symptoms based on factors and conditions related to EDS. For example, specific bodily positions were associated with cognition in PoTS (Maier et al. 2023). Understanding which of these factors plays a role would allow us to base treatment recommendations on addressing the underlying causes of cognitive difficulties, and not solely on improving cognitive skills or strategies.

Significant differences were found for measures of mood, pain, fatigue, cognitive difficulty, and the ability to participate socially. People with hEDS endorsed more symptoms of depression, pain, fatigue, cognitive difficulty, and difficulty with social participation. Given what is known about hEDS and its associated symptoms, this was not unexpected. In fact, previous research has found similar evidence for such symptoms (Kennedy et al. 2022). These symptoms appear to be targets for treatment. In fact, a systematic review of interventions to address pain, fatigue, mood symptoms, and quality of life in people with hypermobility and EDS found that multidisciplinary approaches incorporating both psychological and physical interventions aimed at addressing pain and physical disability were the most effective (Clark et al. 2024).

Finally, we were interested in factors that may relate to subjective cognitive difficulty. To address this, we examined a larger group of individuals with hEDS to examine the correlates of subjective cognitive difficulty. We were interested in whether subjective cognitive difficulty correlated with objective cognition, other subjective measures (anxiety, depression, pain, and fatigue), or both. We found that worse subjective cognition correlated moderately with objective cognitive measures of working memory, visuospatial construction, and verbal fluency set‐shifting. This finding suggests that despite not detecting general cognitive impairment, individuals with hEDS self‐recognize cognitive difficulties and report them. This highlights the clinical importance of recognizing and validating subjective cognitive concerns, in the context of globally non‐impaired objective cognition, as potential avenues for treatment.

Worse subjective cognition was also moderately associated with levels of depression, anxiety, fatigue, and pain. Worse subjective cognition was strongly correlated with difficulty in social participation. Similarly, other recent research on post‐COVID syndrome found associations between subjective cognition and fatigue, anxiety, and depressive symptoms (Delgado‐Alonso et al. 2022). It is possible that believing or experiencing that one's cognition is worse produces anxiety and fatigue. It is also possible that more generalized fatigue, anxiety, and depressive symptoms result in more subjective cognitive dysfunction.

Limitations of this study include its small sample size. A larger sample of people with hEDS would incorporate a greater range of clinical manifestations of the disorder. Additionally, some controversy over the use of neuropsychological assessment via remote tools such as Zoom exists. However, data often supports its use, and as this method was utilized for all participants, no confound was introduced (Brearly et al. 2017). Furthermore, it may be argued that within the population of hEDS, allowing individuals to remain in the comfort of their own spaces may be advantageous.

In conclusion, given our finding that overall cognition was not statistically significantly different between groups, with the exception of delayed verbal memory, this provides some reassuring evidence that for most individuals with hEDS, general cognitive ability, at its best, is likely within normal parameters and not severely affected in a global or stable manner. However, our correlational analysis highlights the need to consider subjective concerns in the context of globally normal performance. We must consider the fluctuating nature of cognition, especially in the context of a multifaceted disorder that often generates pain and commonly fluctuating autonomic nervous system dysfunction. For instance, during an episode of heightened pain or dysautonomia, cognition may be negatively impacted, but in a temporary manner. Therefore, addressing such symptoms may positively impact cognition.

Finally, as subjective cognition was associated with a number of objective cognitive variables, direct cognitive treatment targets may include visuospatial planning and construction, cognitive set‐shifting, and working memory. From a treatment perspective, cognitive interventions aimed at compensating for transient fluctuations may also be a useful tool for managing cognition in people with hEDS. Clinical trials of cognitive interventions for this population are needed.

## Author Contributions

**Amber Sousa**: conceptualization, investigation, writing – original draft, methodology, writing – review and editing, supervision. **Min‐Kyung Jung**: methodology, writing – review and editing, formal analysis. **Arline Allera**: project administration. **Bernadette Riley**: conceptualization, writing – original draft, resources.

## Conflicts of Interest

The authors declare no conflicts of interest.

### Peer Review

The peer review history for this article is available at https://publons.com/publon/10.1002/brb3.70603

## Data Availability

The data that support the findings of this study are available from the corresponding author upon reasonable request.
